# Factors with the Highest Impact on Road Traffic Deaths in Iran; an Ecological Study

**Published:** 2019-07-16

**Authors:** Alireza Razzaghi, Hamid Soori, Amir Kavousi, Alireza Abadi, Ardeshir Khosravi

**Affiliations:** 1 Safety Promotion and Injury Prevention Research Center, Shahid Beheshti University of Medical Sciences, Tehran, Iran. (Email: alirezarazzaghi_21@yahoo.com, ORCID id: 0000-0003-1874-6364 ); 2 Safety Promotion and Injury Prevention Research Center, Shahid Beheshti University of Medical Sciences, Tehran, Iran. (Email: Hsoori@yahoo.com, ORCID id: 0000-0002-3775-1831); 3 Safety Promotion and Injury Prevention Research Center, Department of Epidemiology, School of Public Health and Safety, Shahid Beheshti University of Medical Sciences, Tehran, Iran. (Email: kavousi@sbmu.ac.ir, ORCID id: 0000-0003-3922-0564); 4 Department of Community Medicine, Faculty of Medicine, Shahid Beheshti University of Medical Sciences, Tehran, Iran.; 5 Social Department of Health Research Center, Shahid Beheshti University of Medical Sciences, Tehran, Iran; 6 Department of Statistics and Informatics, Iranian Ministry of Health and Medical Education, Tehran, Iran. (Email: ardeshir.khosravi@gmail.com, ORCID id: 0000-0003-2963-0674)

**Keywords:** Death, accidents, traffic, mortality, multiple trauma

## Abstract

**Introduction:**

The largest proportion of road traffic deaths (RTDs) happen in Low and Middle Income Countries (LMICs). The efforts for decreasing RTDs can be successful if there is precise information about its related risk factors. This study aimed to determine economic, population, road, and vehicle factors with the highest impacts on RTDs in Iran.

**Methods:**

This is an ecological study, which has been done using covariates including: the population density, economic growth, urbanization, distance traveled (km) in 100 thousand people, the length of urban roads, the length of rural roads and the Vehicle per 1000 population for each province of Iran in 2015. The covariates considered had been gathered from different sources and to determine which one of the covariates has an effect on RTDs, the Negative Binomial (NB) regression model was used.

**Results:**

The mean number of RTDs per 100000 population was 474 ± 70.59 in 2015. The highest and lowest rates of death belonged to Fars and Qom provinces, respectively. The results of the univariate model showed the population density as the only covariate of RTDs (p=0.001). Also, among other covariates, GDP was the only variable with a p-value equal to 0.2. In the multivariate NB model, it was seen that the population density (p=0.001), and GDP (p=0.02) significantly correlated with RTDs. For a unit (Million Rial) increase in the GDP of the province, the number of deaths decreased by as much as 0.0014. In addition, for a unit increase in population density, the number of deaths went up by as much as 30.

**Conclusion:**

Population density and GDP had positive and negative effects on the number of fatal road traffic injuries, respectively. By considering these factors in presentational and controlling programs on road traffic injuries, it is possible to decrease the RTDs.

## Introduction

Road traffic crashes (RTCs) are one of the main causes of death in all ages, especially among the 15-29 year-old people all over the world.  The cost of RTCs is approximately 3% of Gross Domestic Product (GDP), which rises to 5% in Low and Middle Income Countries (LMICs).  The increasing trend of RTCs is higher among the countries, which experience the rapid growth of population, urbanization, and motorization (1, 2). A high proportion of road traffic deaths (RTDs) happen in LMICs. There is a rapid increase in income and economic development in LMICs, which causes rapid change and motorization. However, the issues of road safety, related infrastructure development, safety improvement of vehicles, and changing the effective policies are not in accordance with economic changes, urbanization, and motorization, which  leads to manifestation of many problems related to road safety (1, 3). 

In high-income countries, road safety system has managed well in accordance with motorization and economic growth. In these countries, actions such as developing the safer roads, safer vehicles, and effective road safety management system have led to a significant reduction in RTDs (1, 2).

According to international reports, for the first time in history, the global urban living population exceeded 50% of the total population in 2007 and it is constantly rising. By 2020, about 70% of the world population will live in urban areas (4). The world is rapidly urbanizing with extensive changes in population, sustainable mobility feature and the effects of intelligent electronic systems on road safety (1, 4). In some countries such as Iran, the rate of urbanization is higher than economic growth (5). However, effective efforts have not been made to improve safety in the road network and vehicles, in spite of increasing the number of vehicles and the length of roads (1, 6). So in some provinces in Iran, the large number of vehicles and increasing travels in and out of cities make them prone to RTCs (7). In Iran, evaluating the condition of road safety, using the Road Safety Development Index (RSDI), showed that they are not in a good condition regarding road network safety despite little improvements (8). Meanwhile, road network is used for more than 90% of total national shippings in Iran (9).

**Table 1:** Distribution of the studied variables among provinces

Province	GDP (Million Rials)	Traveling (km) per 100000 population	Population density (2015)	Urbanization rate (Percent)	Number of RTD	RTD rate per 100000 population (Adjusted)	Vehicle per 1000 population	Rural Roads (Km)	Urban and Suburban Roads (Km)
**Country**	6225659738	59.55	1.00	69.76	14716		19.22		
**East Azerbaijan**	207139439	42.61	0.0490	71.9	1007	26.26	10.96	6203	3464
**West Azerbaijan**	125717289	42.70	0.0408	65.4	727	22.42	12.42	5107	2948
**Ardabil**	57913670	58.29	0.0160	68.2	179	13.92	11.21	3711	1599
**Isfahan**	85400000	71.83	0.0642	88	723	14.31	21.06	4517	5414
**Alborz**	416864342	43.01	0.0335	92.6	252	9.71	3.95	907	393
**Ilam**	67161398	214.08	0.0072	68.1	155	26.67	14.41	1425	1482
**Bushehr**	212663477	66.10	0.0143	71.9	309	27.51	27.63	1894	2121
**Tehran**	1436431500	69.59	0.1652	93.9	233	11.83	34.37	1589	983
**Chaharmahal and Bakhtiari**	40099640	55.87	0.0118	64.1	182	19.50	13.95	1667	1296
**South Khorasan**	28958350	116.42	0.0096	59	198	25.74	11.86	6686	4455
**Razavi Khorasan**	331292272	97.58	0.0803	73.1	1598	25.15	19.60	7044	6412
**North Khorasan**	34956332	59.83	0.0109	56.1	190	20.90	16.11	2322	1359
**Khuzestan**	836240240	40.66	0.0592	75.5	822	17.16	20.23	8477	5276
**Zanjan**	52830070	34.12	0.0132	67.3	207	19.56	14.03	3590	1658
**Semnan**	55759341	58.66	0.0087	79.8	161	23.92	15.25	1434	1600
**Sistan and Baluchestan**	75230327	63.88	0.0345	48.5	741	26.54	9.69	7401	8010
**Fars**	262027801	69.99	0.0607	70.1	1608	33.62	22.20	8522	7430
**Qazvin**	84992827	30.42	0.0159	74.8	261	20.88	13.93	3239	1341
**Qom**	59519554	56.05	0.0159	95.2	54	4.37	18.91	703	678
**Kurdistan**	60784463	44.87	0.0200	70.8	428	27.88	9.83	4218	1819
**Kerman**	164052960	40.58	0.0395	58.7	852	27.33	16.67	6494	5692
**Kermanshah**	106086048	51.30	0.0247	75.2	484	24.48	11.49	4364	2841
**Kohgiluyeh and Boyer-Ahmad**	143413674	31.90	0.0088	55.7	116	16.50	11.31	2868	1512
**Golestan**	70512931	48.59	0.0234	53.3	444	23.33	16.06	3103	1231
**Gilan**	126890610	42.69	0.0319	63.3	355	13.95	13.27	6445	1891
**Lorestan**	70281385	50.54	0.0222	64.5	360	19.82	14.41	5177	1856
**Mazandaran**	202791471	33.23	0.0410	57.8	773	24.29	14.86	4769	2332
**Markazi**	125424307	42.35	0.0180	76.9	270	18.36	16.03	3608	1845
**Hormozgan**	132781740	43.74	0.0219	54.7	420	24.56	23.06	5341	2897
**Hamadan**	88881887	61.07	0.0220	63.1	433	24.12	16.01	2991	2036
**Yazd**	108536644	63.60	0.0140	85.3	174	15.97	31.04	2580	2295

RTD: Road Traffic Deaths

**Table 2:** The results of univariate analysis using the Negative binomial regression model

95% CI		P-value	SE	Coefficient	Variables
Upper	Low				
**32.75**	11.37	0.001	5.45	22.06	**Population Ratio**
**0.013**	-0.034	0.3	0.012	-0.01	**Urbanization Ratio**
**0.002**	-0.0006	0.2	0.0007	0.00077	**Gross Domestic Product**
**0.06**	-0.03	0.5	0.024	0.013	**Vehicle** 1
**0.003**	-0.03	0.7	0.00004	0.0002	**Urban Road** 2
**0.0003**	-0.002	0.6	0.00003	0.0002	**Rural Road** 3
**0.009**	-0.01	0.8	0.005	-0.0007	**Passenger traveler ** 4

SE: standard error, CI: confidence interval.

1 . Vehicle per 1000 population,

2 Urban Road (km) in each Province,

3 Rural road (km) in each Province,

4 adjusted per/for population.

**Table 3:** The results of multivariate analysis of Negative binomial model

Variables	Coefficient	SE	P-value	95% CI	
				Low	Upper
Population Ratio	30.48	6.36	0.001	17.48	43.48
Gross Domestic Product	-0.0014	0.0006	0.02	-0.0026	-0.00019
Constant	4.32	0.19	0.001	4.95	5.70
In alpha	-1.18	0.24			
Alpha	0.305	0.074			

SE: standard error, CI: confidence interval.

It is expected that RTDs will impose a heavy cost on communities if effective efforts are not made  (10). The governments can make effective efforts to decrease RTCs only if there are valid and reliable data regarding road traffic injuries and deaths (11). In many LMICs, there are no accurate epidemiologic data on RTCs. Using statistical methods can be helpful for determining the factors affecting injuries or deaths (12, 13).

According to global status report on road safety 2018, the estimated rate of road traffic deaths per 100000 populations is 20.5 around the world (1). There is no information on the risk factors of RTD in Iran. This study was conducted to determine factors with the highest impacts on RTDs in economic, population, urbanization, length of roads, and vehicle per 1000 population categories using count regression models. 

## Methods

***Study Design and Setting***

This is an ecological study, which was carried out with the aim of modeling RTDs by studying population density, economic growth, urbanization rate, number of travels, the length of roads, and the number of the vehicles per 1000 population as covariates in all provinces (31 provinces) of Iran using the data of 2015.

***Data Gathering***

In this study, the considered covariates were gathered from different sources. The statistics of RTDs, as a dependent variable in count regression models, were obtained from the Ministry of Health and Medical Education (MOHME). In Iran, registering and collecting the vital data is done by different organizations such as; the National Organization for Civil Registration (NOCR) (as the governmental system that records the vital events), the Iranian forensic Medicine Organization (as a reference point for unnatural deaths), the medical council (as a non-governmental organization for registering all health care professionals), municipalities (as an organization responsible for cemetery in rural and urban areas), and ministry of health and medical education (14). The national reports of World Health Organization in RTDs are prepared by forensic medicine organization (1, 2). However, MOHME is the only registration system, which is based on the International Classification of Disease (ICD) standards. According to the findings of a study in 2009 in Iran, the coverage rate of MOHME registration system is  nearly complete (15). 

The explanatory variables in this study included: urbanization rate in each province (percent), road length (km), Gross domestic product (GDP) (as an economic factor), population density, the number of vehicles per 1000 population, and the distance traveled per 100000 population (km).  

Iran is sub-divided into thirty-one provinces and all data were in the province level. The GDP information of each province was obtained from Tehran Chamber of Commerce, Industries, and Agriculture in 2011 (16). The population of provinces and their urbanization rate were gathered from population census, which has done by the statistical center of Iran (17). The distance (km) traveled per 100000 population by different vehicles was obtained from the information technology office at the Ministry of Road and Transportation (9). The number of registered vehicles was obtained from the Law Enforcement Force of Iran, statistical office (18). The length of road data in each province was obtained from the statistical center of Iran, the transportation sector statistic (17).

***Statistical Analysis***

At first, the Poisson distribution was assessed. In the Poisson model, the mean and the variance should be equal. An over-dispersion in data was found using test of over-dispersion parameter alpha by running the same model using negative binomial distribution. The parameter alpha value equals to 0.305. This strongly suggests that alpha is non-zero and the negative binomial model is more appropriate than the Poisson model (19). The analysis was done in two steps using univariate and multivariate models. The variables with a p-value of less than 0.2 in univariate analysis, entered the multivariate regression model (20). Finally, factors affecting RTDs and their effect sizes were identified. STATA software, edition 14, was used for analyzing the data.

## Results

The results of the study showed that the mean number of RTDs per 100000 population was 474 ± 70.59 in 2015. The highest and lowest rates of death per 100000 population according to Poisson model were related to Fars and Qom provinces, respectively. 

The highest rate of GDP belonged to Tehran, Khuzestan, Isfahan, and Razavi Khorasan provinces. The highest and lowest traveled distances per 100000 people (km) were seen in Ilam and Qazvin provinces, respectively. The population density was highest in Tehran and lowest in Kohgiluyeh and Boyer-Ahmad provinces. The overall urbanization rate in the country (all provinces) was 69.76%. Qom, Tehran, and Alborz had the highest rates of urbanization, respectively. Also, the lowest rates of urbanization were seen in Sistan and Baluchestan, Golestan, and Hormozgan provinces. The highest number of vehicles per 1000 people was observed in Tehran and the lowest in Sistan and Baluchestan. The longest rural roads belonged to Fars, Khuzestan, and Razavi Khorasan, respectively. Also, the shortest rural roads belonged to Qom, Alborz, and Ilam, respectively. About the urban and suburban regions, the highest roads belonged to Sistan and Baluchestan, Fars, and Khorasan Razavi, respectively (table 1).  

The results of univariate analysis using negative binomial model is shown in table 2. The results of the univariate model showed population density as the only covariate of RTDs (p=0.001). Also, among other covariates, GDP was the only variable with a p-value equal to 0.2. 

So, population density and GDP were the covariates selected to enter multivariate negative binomial model. In the multivariate NB model, it was seen that population density (p=0.001), and GDP (p=0.02) both significantly correlated with RTDs (table 3). The value of the β parameter for GDP was equal to -0.0014. In other words, for a unit (Million Rial) increase in the GDP of the province, the number of deaths decreases by as much as 0.0014. Also, the value of the β parameter for the population density was equal to 30.48. This value means that, for a unit increase in population density, the number of deaths rises by as much as 30.

## Discussion

The findings of NB model showed that the effects of population density and GDP on RTD were statistically significant.

Population is one of the factors affecting RTDs. In the Global Status Report on Road Safety (GSRRS) in 2018, the mortality of RTCs was estimated using NB model. In this report, the covariate of population was introduced as an effective factor in RTCs (1).

Some issues emerged following population growth such as: high density of population in cities (21), increase in the number of vehicles, changes in the population demographics, and changes in transportation (22). In LMICs, there is rapid growth in urban areas regardless of related infrastructure and facilities. This issue leads to manifestation of some road traffic related problems including: property damage, injuries, and deaths (23, 24). In Iran, the population has been growing in recent decades and there has been an extensive migration from rural areas to urban areas (7). It should be noted that population growth does not cause an increase in RTCs and their related deaths in all countries. According to GSRRS of WHO in 2018, the number of deaths in Germany (with a population of 81914672), which has a population similar to Iran (with a population of 77447169), was about one fifth compared to Iran (3206 versus 15932) in 2016 (1). Therefore, Germany and Iran have a similar population, but the number of deaths is not the same. One of the reasons for this difference is discrepancy between rapid growth of population and capacity building in the transportation system (21)(25). While in many high-income countries, the increase in population has been followed by effective changes in the transportation system. 

For example, the increasing trend of the population has caused the shift from private motorized transport to public transport, or has led to making the infrastructure for cycling or walking instead of using a motorized vehicle. Moreover, the rapid changes in vehicle technologies and their improvement by applying intelligent systems have caused improvement in the safety and prevention of crashes and related deaths (26). Along with population growth, there are some changes in demographic characteristics. For example, in many countries the elderly population has an increasing trend. According to WHO report in 2015, the proportion of elderly people in Iran (people 60 years or above) will double during 2015-2030 (27). The findings of other studies show that the most important injury among the elderly people is road traffic injuries, which has the highest incidence, death rate and Disability Adjusted Life Years (DALY) among them (28).

GDP (as an economic factor) was the second factor affecting RTDs in this study. Most studies in this area have used economic indicators such as GDP (29). It was shown that with a raise in GDP, the number of deaths has decreased in provinces. The findings of earlier studies showed that road traffic deaths will increase with launch of development. The rate of RTDs will begin to decrease when exceeding a threshold level in economic status (30, 31). In the early stages, economic growth leads to an increase in vehicles and this condition leads to increase in their related injuries and deaths. This is more important for LMICs, which are mostly in the early stages of economic developing. The findings of a study in 1975-1988 showed that the rate of road traffic deaths in Malaysia and Colombia had increased; yet, it had decreased in high-income countries by as much as 25-50% (32). The results of a study in Brazil in 2008 showed that in the previous decade the north and north-east areas with low GDP had higher rates of death in comparison with other areas with high GDP (33).

In Iran, the high rate of road traffic deaths correlates with the number of vehicles. The number of vehicles has raised following economic growth, which is in its early stage in Iran (5). According to reports of statistical center of Iran, the rate of motorization (the number of vehicles per 1000 population) was higher than the economic growth rate during the years 1971-2009 (5). In low and middle-income countries, failure to balance economic growth with the motorization can play a role in increasing the incidence of road traffic crashes. However, in Iran the rate of road traffic deaths has had a decreasing trend in 2007-2018 (34). So the decreasing effect of GDP on RTCs can be explained considering that economic growth has led to an improvement in road safety and raise in investment in transport infrastructure and this has ultimately led to a reduction in RTDs (5, 35). 

1. **Strengths and Limitations**

 One of the strengths of this study is that it was implemented at the national level and included provincial comparison. On the other hand, this is an ecological study and this should be noted in the interpretation of results. In an ecological study, the ecological inference fallacy occurs if this will not be considered in the interpretation of ecological level data to the individual level. One of the limitations of this study could be the possible information bias in RTD data obtained from the MOHME. 

## Conclusion:

The covariate population density increases the fatal road traffic injuries and Gross Domestic Production decreases that. By considering these factors in presentational and controlling programs done on road traffic injuries, it is possible to further decrease road traffic deaths. 

## References

[1] World Health Organization (2018). Global status report on road safety.

[2] World Health Organization (2015). Global status report on road safety.

[3] Road Deaths (2018). Injuries Hold Back Economic Growth in Developing Countries.

[4] City BL, Assessment E (2010). Urbanization and health. Bulletin of the World Health Organization.

[5] Mehrgan N, Gholizadeh A, Mohammadi F (2012). Road traffic accident in a economic analysis[Persian]. Transportation Engenering..

[6] World Health Organization (2010). Data systems: a road safety manual for decision-makers and practitioners.

[7] Seifdini F, Mansourian H, Pour-Ahmad A, Darvishzadeh R (2014). Processes and Patterns of Urbanization in Iran [Persian]. Urban and Regional Studies and Research.

[8] Jafari MR, Vosoughi S, Abadi M, Khandan M (2017). Comparing road safety conditions in Iran with ten Southeast Asian countries using road safety index. Iran Occupational Health.

[9] (2015). Road transportation and road organization of the country; IRAN [Persian].

[10] Zhang X, Hongyan Y, Guoqing H, Mengjing C, Yue G, Xiang H (2013). Basic characteristics of road traffic deaths in China. Iranian journal of public health.

[11] Bhalla K, Sharaz S, Abraham J, Bartels D, Yeh P-H (2011). Road Injuries in 18 Countries: Methods, data sources and estimates of the national incidence of road injuries.

[12] Mohan D (1984). Accidental death and disability in India: a stocktaking. Accident Analysis & Prevention.

[13] Soori H, Khorasani-Zavareh D (2019). Road traffic injuries measures in the Eastern Mediterranean Region: findings from the Global Status Report on Road Safety–2015. Journal of injury and violence research.

[14] Aalaei H, Amini F, PakNia B, Jafari M, Milani M, Farrokhi B (2014). Guidance of filling and classification of causes of deaths [Persian]: Iran Ministry of Health.

[15] Bhalla K, Shahraz S, Bartels D, Abraham J (2009). Methods for developing country level estimates of the incidence of deaths and non-fatal injuries from road traffic crashes. International journal of injury control and safety promotion.

[16] (2014). Reviewing and ranking of GDP and value added Economic activities of the provinces of the country in the year 2011 [Persian].

[17] (2015). Iran Statistical Year Book.

[18] (2015). IRI Police Department.

[19] Park ES, Lord D (2007). Multivariate Poisson-lognormal models for jointly modeling crash frequency by severity. Transportation Research Record.

[20] Hongyue W, Jing P, Bokai W, Xiang L, Julia ZZ, Kejia W (2017). Inconsistency between univariate and multiple logistic regressions. Shanghai archives of psychiatry.

[21] Henderson V (2002). Urbanization in developing countries. The World Bank Research Observer.

[22] Hakkert AS, Gitelman V (2014). Thinking about the history of road safety research: Past achievements and future challenges. Transportation research part F: traffic psychology and behaviour.

[23] Iamtrakul P, Hokao K (2012). The study of urbanization patterns and their impacts on road safety. Lowland Technology International.

[24] Peden M, Scurfield R, Sleet D, Mohan D, Hyder AA, Jarawan E (2004). World report on road traffic injury prevention.

[25] Desa U (2014). World urbanization prospects, the 2011 revision. Population Division, Department of Economic and Social Affairs, United Nations Secretariat.

[26] Torok A, Derenda T, Zanne M, Zoldy M (2018). Automatization in road transport: a review. Production Engineering Archives.

[27] Organization WH (2015). World report on ageing and health: World Health Organization.

[28] Naghavi M, Abolhassani F, Pourmalek F, Jafari N, Moradi Lakeh M, Eshrati B (2008). The burden of disease and injury in Iran in the year 2003. Iranian Journal of epidemiology.

[29] MOHAMMAD AS, ASEFZADEH S, MOHEBBIFAR R, MONTAZERI A (2012). Survey of Human Development Index (HDI) In Iran and Selected Countries.

[30] Anbarci N, Escaleras M, Register C (2006). Traffic fatalities and public sector corruption. Kyklos.

[31] Bishai D, Quresh A, James P, Ghaffar A (2006). National road casualties and economic development. Health economics.

[32] Kopits E, Cropper M (2003). Traffic fatalities and economic growth.

[33] Chandran A, Sousa TRV, Guo Y, Bishai D, Pechansky F, Team TVNTE (2012). Road traffic deaths in Brazil: rising trends in pedestrian and motorcycle occupant deaths. Traffic injury prevention.

[34] Statistical Report of Road Traffic Deaths (2018). Iranian Legal Medicine Organization.

[35] van Beeck EF, Borsboom GJ, Mackenbach JP (2000). Economic development and traffic accident mortality in the industrialized world, 1962–1990. International journal of epidemiology.

